# Digital sufficiency: conceptual considerations for ICTs on a finite planet

**DOI:** 10.1007/s12243-022-00914-x

**Published:** 2022-05-12

**Authors:** Tilman Santarius, Jan C. T. Bieser, Vivian Frick, Mattias Höjer, Maike Gossen, Lorenz M. Hilty, Eva Kern, Johanna Pohl, Friederike Rohde, Steffen Lange

**Affiliations:** 1 Department for Social Transformation and Sustainable Digitalization, Technical University of Berlin, Berlin, Germany; 2 Institute for Ecological Economy Research, Berlin, Germany; 3 Einstein Centre Digital Future, Berlin, Germany; 4 Department of Informatics, University of Zurich, Zurich, Switzerland; 5 Department of Sustainable Development, Environmental Science and Engineering, KTH Royal Institute of Technology, Stockholm, Sweden; 6 Technology and Society Lab, Empa Materials Science and Technology, St. Gallen, Switzerland; 7 Environmental Campus Birkenfeld, Birkenfeld, Germany; 8 Resource Economics Group, Humboldt-Universität Zu Berlin, Berlin, Germany

**Keywords:** Green IT, ICT for sustainability, Sustainable software, Sustainable production and consumption, Rebound effects, Economic growth, Degrowth

## Abstract

ICT hold significant potential to increase resource and energy efficiencies and contribute to a circular economy. Yet unresolved is whether the aggregated net effect of ICT overall mitigates or aggravates environmental burdens. While the savings potentials have been explored, drivers that prevent these and possible counter measures have not been researched thoroughly. The concept digital sufficiency constitutes a basis to understand how ICT can become part of the essential environmental transformation. Digital sufficiency consists of four dimensions, each suggesting a set of strategies and policy proposals: (a) hardware sufficiency, which aims for fewer devices needing to be produced and their absolute energy demand being kept to the lowest level possible to perform the desired tasks; (b) software sufficiency, which covers ensuring that data traffic and hardware utilization during application are kept as low as possible; (c) user sufficiency, which strives for users applying digital devices frugally and using ICT in a way that promotes sustainable lifestyles; and (d) economic sufficiency, which aspires to digitalization supporting a transition to an economy characterized not by economic growth as the primary goal but by sufficient production and consumption within planetary boundaries. The policies for hardware and software sufficiency are relatively easily conceivable and executable. Policies for user and economic sufficiency are politically more difficult to implement and relate strongly to policies for environmental transformation in general. This article argues for comprehensive policies for digital sufficiency, which are indispensible if ICT are to play a beneficial role in overall environmental transformation.

## Introduction

The discourse on the environmental sustainability of using information and communication technologies (ICT) has become increasingly well founded, complex, and interdisciplinary. Nevertheless, large research gaps remain regarding both empirical and conceptual and theoretical knowledge (for an overview, see [1, 2]). This paper mainly addresses the following three research gaps.

1. Various studies on ICT and environmental sustainability have identified the potential of ICT to reduce energy and resource inputs but do not consider important trends that run counter to that potential and, eventually, limit ICT positive contributions (e.g., [3–6]). For example, ICT-borne efficiency improvements may cause rebound effects, which countervail parts or all of the savings potential [7–9]. An increasing number of publications analyze ICT-borne rebound effects [9–15]. However, an overarching strategy to cope with rebound effects and with further undesired effects of ICT use (e.g., induction effects) has yet to be presented.
2. While many studies on ICT and environmental sustainability provide valuable scientific analyses, they do not develop comprehensive policies and measures addressing all relevant societal actors. For example, several studies focus on sustainability of software engineering but do not investigate the combination of both hard- and software and its implications for policy-making [16–19]. Other studies focus on a certain analytical level, e.g., by differentiating between first-, second-, and third-order impacts, but fail to discuss policy solutions that systematically address all those levels ([1, 20, 21]).
3. Most studies on ICT and environmental sustainability do not account for existing sustainability strategies. Those strategies suggest that a combination of efficiency, consistency, and sufficiency strategies is most effective in realizing absolute savings [22–24]. Hilty et al. propose that sufficiency strategies are particularly suited to addressing ICT-borne rebound and induction effects [25]. However, sufficiency strategies have not yet been further specified in the context of ICT.

Recognizing the research gaps on sufficiency strategies for ICT use and the lack of comprehensive and actor-specific policy recommendations for environmentally sustainable ICT use, including how to address ICT-borne rebound effects, this paper develops a comprehensive “digital sufficiency” concept. The concept consists of four dimensions: (a) hardware sufficiency, (b) software sufficiency, (c) user sufficiency, and (d) economic sufficiency. Along these four dimensions, we derive and discuss a comprehensive set of policies for environmentally sustainable ICT use that involves all major groups of actors: policymakers, individual users, software developers, the business sector, and civil society. By doing so, we advance the conceptual knowledge on environmental sustainability aspects of ICT use and provide concrete guidance for practitioners wishing to align ICT use with environmental protection.

The article is structured as follows. In Section 2, we define sufficiency, link it to the “ICT for Sustainability” debate, and reason why digital sufficiency is an expedient strategy to ensure that ICT contribute positively to environmental sustainability. In Sections. 3 and 4, we define our approach and the four sub-dimensions of digital sufficiency. For each dimension, we identify challenges that prevent resource and energy savings and suggest policy measures to overcome them. In Section 5, we summarize our findings, highlight interrelationships of the four dimensions of digital sufficiency, and conclude with a self-critical discussion on environmental effectiveness and social acceptance of the suggested policy measures.

## State of literature 

### Consistency, efficiency, sufficiency

Over the past decades of environmental sustainability research, three overarching strategies have been developed to reduce environmental burdens: consistency, efficiency, and sufficiency [23, 24]. The strategy of consistency aims at “doing things better,” namely, closing material and nutrient cycles and bringing cycles of industrial production and consumption in line with natural cycles, including those of water, air, climate, or soil recovery. The “circular economy” [26–28] or the “cradle-to-cradle” concept [29] are types of consistency strategies. The strategy of efficiency aims at “doing more with less,” namely, reducing resource and energy inputs per unit of service or product. Innumerable articles and studies have been published in favor of increasing energy and resource efficiencies in different sectors and domains (e.g., [30, 31]). The strategy of sufficiency focuses on an absolute reduction of resource and energy demand while maintaining, or even improving, immaterial living conditions and a “good life” for all [32].

Various definitions of sufficiency can be found. Most of them understand sufficiency as avoiding overconsumption while reducing the use of scarce natural resources and fossil fuel-based energy [1–3]. Traditionally, most literature on sufficiency has focused on individual consumer behavior, e.g., rethinking personal needs and avoiding excessive consumer behavior [35, 36]. Sufficiency-orientated behavior includes absolute reductions of consumption, modal shifts to more resource-efficient transport modes, product lifetime extension, and sharing practices [35, 37]. Related terms and concepts have been discussed as “voluntary simplicity,” “frugality,” “downshifting,” “anti-consumption,” “minimalism,” or “slow consumption” (e.g., [38–40]). Despite the focus on individual behavior, several authors have also applied the concept of sufficiency to the production side (e.g., for sufficiency-oriented marketing strategies) and to the overall economic level of activity [41–44]. In addition, much of what is currently discussed as “postgrowth” or “degrowth” economics has a strong affinity to sufficiency—both because politics for degrowth always include sufficiency proposals [45–48], and because the term “sufficiency economy” describes concepts for the macro-level that are similar to concepts for a postgrowth economy [44, 49].

We define sufficiency as any strategy that directly aims at decreasing the absolute level of resource and energy use by reducing the levels of production and consumption. Sufficiency necessarily involves reflecting upon existing individual and societal needs, attitudes, and beliefs. And it requires changes in consumption practices as well as in production structures, infrastructures, and existing political incentives that favor consumerism and conventional economic growth.

Current political efforts to reduce resource demand and emissions to sustainable levels often pursue technology-based approaches that focus on efficiency and consistency, and often neglect sufficiency [50, 51]. For instance, with Sustainable Development Goal No. 12, the global community agrees on the principle of “doing more and better with less” [52], which refers to efficiency strategies to achieve a win–win situation for economy and ecology. Yet, both efficiency and consistency strategies ignore rebound, induction, and growth effects and, hence, may fail to deliver significant savings, as any relative reduction of inputs can be outplayed by an absolute increase of output [53, 54]. Therefore, without neglecting the valuable contributions of efficiency and consistency strategies, several authors suggest that those strategies should be accompanied—if not guided—by a sufficiency strategy that ensures absolute reductions in resource and emissions intensities [55–57]. Furthermore, several authors overcome the ill fortune that sufficiency is sometimes mistakenly associated with attitudes such as abstinence or renouncement by suggesting that the sufficiency strategy can benefit both the environment and the quality of life [32, 33, 58, 59].

## Environmental impacts of ICT and reasons for digital sufficiency

ICT environmental effects have been under discussion for more than a decade [1, 21, 60–64]. A few studies have attempted to calculate ICT aggregate environmental impacts throughout all sectors [5, 6, 65]. However, those studies cannot claim to exhaustively cover all global impacts of ICT, and methodological developments for a comprehensive and consistent synopsis are still in their infancy [6]. For certain sectors or fields of applications, a large number of studies have been conducted—documented, for instance, by the growing number of publications in the discourse “ICT for Sustainability” [66]. However, sector-based, micro-level, or case study analyses obviously do not allow for conclusions on whether the aggregated net effect of introducing ICT into society is positive or negative [1, 67, 68].

Over the years, a taxonomy of first-order and higher-order environmental effects has emerged [21]. First-order or “direct effects” relate to producing ICT devices and infrastructures and to the electricity demand from using those digital devices and services. Higher-order or “indirect effects” result from social change associated with applying and using ICT. Hilty [1], Horner et al. [21], and others distinguish between positive higher-order environmental effects, such as substitution or optimization effects, and negative environmental effects, such as rebound or induction effects (see also [11, 69]). Yet, because the introduction of ICT devices and services can be associated with a broad spectrum of behavioral and structural changes in economy and society, the net sum of all higher-order effects are difficult to determine.

The literature identifies four mechanisms that inhibit the realization of positive effects and build the basis for devoting greater attention to sufficiency. First, “direct effects” (first-order) come with manifold environmental problems, including extraction of scarce resources and insufficient environmental standards within the ICT production process [70–72]. Although ICT can contribute to enhancing recycling and circular material flows in other sectors [26], ICT hardware production itself is far from living up to the premise of environmental consistency, which would imply that devices be made of renewable resources or fully recycled materials in a circular economy.

Second, the volume of data storage, processing, and transmission is currently increasing exponentially. For example, data volumes are being particularly driven by big data analytics, artificial intelligence, and online video streaming [73]. Notwithstanding the fact that energy and resource efficiencies of data processing and transmission are constantly increasing (e.g., by the shift from 3 to 4G and eventually 5G mobile networks) and that there is a non-linear relationship between data traffic and environmental footprint [74], past saving potentials have so far been either fully or to a large part outpaced by vastly increasing data volumes [75].

Third, not only hardware, software, and ICT system management have become more efficient, but also the application of ICT in many areas of economic production and consumption has improved various efficiencies, including cost efficiency, energy efficiency, time efficiency, and behavioral efficiency. Such efficiency improvements may help reduce existing environmental burdens [5, 76–78], but at the same time, they also generate rebound effects, which countervail parts of the savings potential, if not all [7, 15, 53]. Even in cases where rebound effects at micro-level are small, many studies have shown that taking into account higher-order rebound effects can be decisive for the aggregate environmental impact [13, 61, 79–81].

Fourth, the introduction of ICT applications opens up new opportunities for production and consumption and, hence, leads to induction effects, which occur if ICT use generates additional energy and resource demand [2, 11, 82]. Overall, ICT-borne rebound and induction effects foster economic growth [83], which eats up potential energy and resource savings from optimization and substitution effects.

Note that these four mechanisms do not necessarily imply a negative aggregate environmental impact of ICT, but they do curtail the contributions derived from efficiency and consistency strategies. Hence, in the following sections, this paper elaborates the concept of digital sufficiency in a way that addresses each one of these four countervailing forces.

To date, only few publications address sufficiency strategies in the context of ICT. Back in 2008, Hilty mentioned the need for sufficiency strategies with regard to ICT [88]. Santarius and Lange were the first to coin the term “digital sufficiency” and develop the concept in their book “Smart Green World” [89]. They present three dimensions: technical sufficiency, data sufficiency, and user sufficiency [2]. This article is based on that work but elaborates on the concept and adds a fourth dimension—economic sufficiency.

The “Shift Project” report has introduced the terms “lean ICT” and “digital sobriety” [90]. Given the overall messages of the report, these terms appear close to the concept of digital sufficiency presented here. However, the report neither clearly defines the terms nor elaborates on what dimensions or elements the concept includes.

## Methodology

The conceptual considerations on digital sufficiency presented in this article have been developed in two specific contexts. First, in 2016 the 6-year research group *Digitalization and Sustainability* (www.sustainable-digitalization.org) was established, aiming “to analyze rebound risks and sufficiency opportunities from digitalization.” Six authors of this paper are members of that group, stemming from different disciplines (psychology, marketing, engineering, sociology/social sciences, economics). This article is based on internal colloquia conducted between 2016 and 2019. The colloquia applied techniques of interdisciplinary co-creation [91, 92] to synthesize conceptual considerations of digital sufficiency that were developed and explored in greater detail within smaller scientific projects and doctoral theses. In addition, three transdisciplinary workshops [93] with representatives from industry, civil society, labor unions, and federal government bodies were conducted to develop “transformation knowledge” and “target knowledge” [93] regarding digital sufficiency. The policy proposals presented here were developed and critically examined in those workshops.

Second, the digital sufficiency concept was further advanced in a workshop consisting of 12 researchers and practitioners at the *7th International Conference on ICT for Sustainability* in Finland in 2019. This workshop integrated perspectives from two epistemic research communities: social sciences addressing issues of sustainable development and technical sciences addressing challenges of digitalization/ICT. The multidisciplinary co-authorship of this paper was set up as an upshot of that workshop and integrates knowledge from the disciplines of the research group with knowledge from the disciplines of informatics, computer sciences, and future studies.

## Digital sufficiency and its four dimensions 

Building on our general definition of sufficiency (Section  2.1.), we define “digital sufficiency” as any strategy aimed at directly or indirectly decreasing the absolute level of resource and energy demand from the production or application of ICT.

Based on the abovementioned four mechanisms related to why ICT currently fall short in delivering environmental gains (see Section 2.2.), we distinguish four dimensions of digital sufficiency: (a) hardware sufficiency, (b) software sufficiency, (c) user sufficiency, (d) economic sufficiency. To elucidate the difference between these four dimensions and corresponding strategies for efficiency and consistency, each of the following subchapters starts by explaining the related efficiency and consistency terms and contrasting those terms with what is meant by sufficiency for the respective dimension.

### Hardware sufficiency

Increasing hardware *efficiency* would aim either at reducing the energy and material per production unit of hardware (e.g., per fabricated smartphone) or at increasing energy efficiency in the use phase to keep relative energy demand per unit of computing power at a minimum. Hardware consistency would aim at eliminating toxic materials in the ICT production process, ideally achieving production cycles with fully renewable or recycled materials, powered by renewable energy.

In contrast, *hardware sufficiency* aims at being able to produce fewer devices, designing devices last for a long time, ensuring that their complexity and resource use do not surpass the purpose they are designed for (“not cracking a nut with a sledge-hammer”), and keeping their absolute energy demand at the lowest level possible to perform the desired tasks.

#### Challenges throughout the lifecycle of ICT hardware

Environmental impacts throughout the life cycle of ICT hardware (production, operation, disposal) are caused by mining raw materials for production, causing production waste and emissions, providing the energy needed in all phases of the life cycle, and by the processes of end-of-life treatment [88, 94]. Production of ICT hardware requires more than 50 chemical elements, including many scarce and toxic metals, the mining of which often has toxic impacts on humans and ecosystems [95]. For end-user devices, the production phase usually accounts for the highest share of lifecycle-wide energy demand and CO2 emissions [72, 94]. Device collection remains a main challenge at the end of product life. Devices from data centers, base stations, etc. are more often recirculated while PCs, tablets, smartphones, and other end-user devices are often stored at home. If collected, removing toxic components and recovering scarce metals remain major issues [96].

Some of these challenges can be addressed by strategies towards greater hardware consistency. But given the limited knowledge on raw material substitutes, basing all hardware production on 100% renewable resources within a reasonable period of time does not seem feasible [97, 98]. Moreover, even under optimum industrial conditions, only a small subset of the materials can be recovered [95] and much ICT hardware enters informal recycling channels from which few elements are recovered, often under hazardous conditions [99].

At the same time, the absolute number of ICT devices is constantly increasing: An increase from 18 billion ICT devices in 2017 to over 27 billion devices in 2022 is expected [100]. Moreover, the acceleration of product cycles increases demand for new hardware and stimulates resource depletion. Functioning hardware is often rendered obsolete by software evolution or even by planned obsolescence [101]. Furthermore, the trend towards the Internet of Things, in which everyday objects are enhanced with ICT hardware, could lead to increased software-induced obsolescence [18], which will affect not only the electronics part but the whole “smart thing” in which it is embedded [88].

During the use phase, impacts are mainly caused by the electricity required to power ICT hardware [1]. The environmental impacts depend on the electricity mix used for operating the hardware, a mix still dominated in many countries by fossil fuels. Especially for data centers, which operate permanently, electricity consumption causes major environmental impacts throughout the whole lifecycle. Besides the amount of data processed (see software sufficiency below), decisive factors for the energy demand of data centers are waste heat recovery, cooling technology, and server utilization [102]. Data center infrastructure and associated environmental impacts are expected to increase significantly in the future [103, 104].

Altogether, such challenges are likely to countervail large parts of the saving potentials achieved by strategies that improve efficiency in hardware production. Undoubtedly, improving the material and energy efficiency of ICT and using electricity from renewable sources can be effective levers to reduce environmental impacts. However, efficiency and consistency strategies alone may not lead to absolute savings [105]—which is why sufficiency strategies are essential.

#### Elements of hardware sufficiency

Extending the useful life of devices reduces the demand for new devices and thus slows down the flow of resources from extraction to waste. If manufacturers design repairable and upgradable devices (e.g., through modular design), these will be able to match demand for computing power for a longer period. Also, purchasing smaller devices (e.g., laptops instead of desktop computers) often reduces impacts as these require less material resources and energy during both production and operation [94].

With regard to end-of-life treatment, improving the recycling systems in terms of collection and recovery rates is also important. To improve recovery, devices need to be designed for it (e.g., through use of screws instead of glue) [95]. However, the goals of reparability and recovery often conflict with the demand for compact and light devices.

#### Policies that promote hardware sufficiency

Policies targeting hardware sufficiency mainly address manufacturers, retailers, purchase departments, and infrastructure providers (e.g., network, data center) and relate to changes in hardware design, purchase, use, and end-of-life treatment. With respect to infrastructures, policies should incentivize shared use among providers to increase utilization, reduce hardware use, and create synergies in building and operation. Beyond long-lasting design and efficiency, network hardware in base stations can also be mutualized in many ways.

Policies for hardware design, which significantly influences the environmental impacts during production, operation, and end-of-life treatment, should focus on four aspects. (1) Developing standards that ensure low environmental impacts during production, e.g., a design directive can require manufactures not only to avoid critical and hazardous materials (consistency) but also to constantly increase the share of recycled materials and reused parts. (2) Legislating design principles for long-lasting hardware, e.g., extending warranty periods and setting minimums (e.g., components need to be replaceable, use of standard interfaces). (3) Enacting energy consumption standards for hardware, e.g., setting standards not for relative energy consumption (efficiency) but for absolute energy consumption of hardware. (4) Developing policies to encourage reuse and recycling, e.g., by setting minimum standards for the rates in recovering metals, which would also benefit manufacturers as soon as the recovery of materials from used devices is cheaper than the mining of raw materials.

To enhance sufficiency in purchasing hardware, first of all, public and private organizations can adopt policies to reduce the number of devices, e.g., through bring-your-own-device (BYOD) strategies or private use of company-owned devices. For those devices still purchased, unbiased information is needed to enable acquisition of devices conforming to sufficiency-oriented hardware design (see above). Manufacturers should voluntarily or mandatorily be transparent about environmental and social production standards (e.g., in sustainability reports), the materials used (e.g., through mandatory “ingredient lists,” as for food), energy demand during operation, and upgrade and repair possibilities.

To foster sufficiency in the use phase, policies should aim at avoiding unnecessary early termination of a hardware’s useful life, e.g., manufacturers should offer repair and upgrade services and provide necessary software updates for operating systems until the end of a device’s physical lifetime. Moreover, hardware companies should change their business models from selling to letting (device-as-a-service), allowing devices that do not meet the requirements of the users to be returned and redistributed to other users after refurbishment. This service would also create incentives for manufacturers to design long-lasting devices as their revenues would be decoupled from the number of devices produced. Moreover, hardware sellers should refrain from marketing faster product lifecycles, e.g., bundled contracts that include a new smartphone after a certain period.

Finally, to improve end-of-life treatment, policies should aim at improving collection and recovery rates, e.g., legislation should make take-back programs mandatory, as in Switzerland (Schweizerischer Bundesrat, 2006). Device-as-a-service models would also increase collection rates. Moreover, hardware flows after take-back have to be overseen to ensure that informal recycling is avoided. Manufacturers should collaborate with formal and informal recycling facilities to improve processes.

Hardware sufficiency can foster user sufficiency (see below) whenever manufacturers empower ICT buyers to improve how resources are used. For instance, hardware producers can support sufficiency-oriented use by granting a “right to repair,” i.e., allow users to hack digital locks on devices in order to repair those devices themselves. Legislation that helps advance open source hardware products would incentivize hardware producers to move in that direction [106].

### Software sufficiency

Increasing software *efficiency* would aim at reducing the demand for electricity and hardware utilization per unit of computing power or data transmission.^1^ In contrast, *software sufficiency* aims at software being developed so that data traffic and hardware utilization during application are as low as possible in absolute terms. Hence, software sufficiency includes strategies that actually reduce data volume and traffic and demand for computing power and that increase the service life of ICT hardware.

#### Challenges of resource-intense data storage, processing, and transmission

In 2017, annual global Internet traffic was estimated at 1.5 Zettabyte (ZB), with IP video (Internet Video and IP VOD/ Managed IP Video) being responsible for 75% [100]. Data traffic has increased rapidly since the early 1990s from 100 GB per day in 1992 to 100 GB per second in 2002 to 46,000 GB per second in 2017 [107]. By 2022, global data traffic is expected to triple to 150,000 GB per second (4.8 ZB per year) [100]. The largest share of IP data traffic takes place within data centers [108], which, together with the networks, account for about half of the sector’s operational electricity demand [76]. The networks’ contribution to that demand is determined by the type of access network (mobile vs. fixed, optical fiber vs. ADSL), bandwidth, utilization factor of network components, and the kind of access device used [103, 109–111].

There are four reasons for the exploding data volume, which also contributes to the increasing energy demand of the ICT sector. First, the number of devices that are online and, more relevant, that are transferring data without human intervention, i.e., within the Internet of Things (IoT), has already vastly increased in past years and is expected to increase further in the coming years [100]. Second, the increased use of data-intensive applications leads to software bloat [112], i.e., software that is too large and resource intensive for its purpose and often leads to well-functioning existing hardware being unnecessarily exchanged for more powerful hardware (see hardware sufficiency). Machine-learning algorithms, in particular deep learning, are an example of particularly data-intensive applications [113, 114]. Third, the Big Data trend [115] contributes to increasing data traffic [108]. Fourth, the increasing use of cloud services can contribute to energy consumption, for instance, if data storage is doubled and data transfers increase overall [108, 116].

Given these challenges, saving potentials from strategies that aim at improving software efficiency are likely to be fully eaten up by high overall growth rates. Note that, while energy consumption through the production and operation of hardware has been relatively well studied, investigations on the effects of software on overall energy consumption of ICT are only just appearing [16, 17, 117].

#### Elements of software sufficiency

To achieve software sufficiency, three parameters must be considered: the characteristics of software products, the amount of data generated, and the technical parameters affecting the energy intensity of ICT infrastructure and devices.

First, software needs to be designed for minimizing electricity and resource demand in the use phase. Software design principles should consider the dimension of software sufficiency right at the beginning of the software lifecycle [118]. One example is the Karlskrona Manifesto for Sustainability Design, which presents a set of principles and commitments such as “Sustainability requires long-term thinking” and “Sustainability applies to both a system and its wider contexts” [19]. Another example is the set of criteria and indicators for sustainable software products developed by Kern et al. [18]. The criteria address default settings towards minimal energy demand, management functionality of hardware capacities to avoid wasteful use, and backward compatibility of software to mitigate obsolescence [18].

Second, the amount of data collected and transmitted must be minimized. Software products should avoid data transfer that is not necessary for a service to provide its intended functionality. For instance, reporting of user behavior or program crashes should not transfer data automatically, and offline use should be possible. During development, the question “For what purpose is the data generated or required?” should be a constant companion. The concept of data sufficiency, e.g., as enshrined in the EU’s General Data Protection Legislation (GDPR), may serve as a guiding principle. Software products using open data standards and open source software make data and source code available and, thus, prevent redundancy. Moreover, they can also help to avoid software-induced obsolescence (see hardware sufficiency above). Additionally, wired or WiFi networks should be preferred over mobile networks [119]. Software should dynamically change to a more energy-saving mode whenever possible [18].

Third, required hardware capacities should be adapted according to the current demand [18], allowing software products to manage and control the hardware capacities they occupy, e.g., by setting parts of the network infrastructure into sleep mode at low utilization rates. In data centers, IT management software can foster minimal energy and resource demand by improving overall processes (Enterprise Resource Planning) and energy consumption (energy analytics) and by implementing the principle of neuromorphic computation [120].

#### Policies that promote software sufficiency

Policies targeting software sufficiency mainly address software developers, data center operators, and cloud service providers.

For software developers, the development and mandatory application of sustainable software design principles [18] can ensure that software is programmed so as to minimize *absolute* energy demand. This programming includes criteria such as default settings towards minimal energy demand, limiting the extent of forced connectivity, applying open standards, or mitigating hardware obsolescence by ensuring backward compatibility [18]. Sustainable interaction design principles should also be applied to website development. These principles would not only minimize the absolute energy consumption of websites but also enable access even with slow Internet connections [121].

Moreover, sufficiency-oriented software design principles also mean reducing the release speed of new software, applications, or frameworks. This includes a separation between security updates and evolutive updates. A sufficiency stand on software would lean towards maintenance/care of existing software, rather than on constant novelty.

Labels for *energy sufficient software* can promote environmentally friendly solutions, create an awareness of sustainability issues among software developers, and help users choose between alternatives. Their implementation can be enhanced by making the label mandatory for public procurement and by extending the European Ecodesign Directive to software products.

A similar approach is also conceivable for data centers. By making the application of IT management software mandatory in data centers, minimal energy and resource demand can be further enhanced. Further, a data center pricing policy that takes into account the actual energy and resource consumption would reward customers who pay attention to strategies that minimize data amount and traffic. In addition, by flexibly managing computation-intensive processes so that they only run when the electricity grid is not highly utilized, data centers can contribute to load balancing at high feed-in rates. These management processes become particularly relevant for renewable energy-powered data centers.

Beyond the approaches dealing with developing and implementing sufficiency-oriented software, data could also be limited directly through limiting the speed of wireless Internet connections, allocating a specific energy budget to the Internet or raising energy prices [122]. However, note that this would shift the burden from software developers onto users and could affect net neutrality. An advertising ban on the Internet could further reduce the absolute Internet data volumes [123]. Other approaches are concerned with limiting broadband capacities [124] or limiting online video resolution [125].

Software sufficiency can contribute to hardware sufficiency if software products are designed to run on older hardware. This design can help avoid having to replace existing hardware. Software sufficiency can also contribute to user sufficiency (see below) if applications actively help users to reduce hardware and energy demand and data traffic. This support can be ensured by various *default settings*, e.g., by strict privacy settings, by providing the minimal resolution appropriate for online video and pictures, by erasing unnecessary data generated during operation, or by disabling further consumption-increasing nudges such as autoplay.

#### User sufficiency

For the sustainability implications of ICT from the user perspective, two aspects need to be distinguished: how are ICT used (number of devices, intensity, duration) and what are ICT used for (purpose, activity).

Related strategies that aim at increasing *efficiency* from the user side include, e.g., buying energy efficient devices or using ICT to organize one’s existing consumption needs with the least possible resources. Strategies that aim at increasing consistency range from sourcing green electricity for ICT devices to searching the Internet for the most environmentally friendly produced goods and services.

In contrast, *user sufficiency* has two aims: users apply digital devices frugally and they use ICT that promote sustainable lifestyles and enable them to reduce their consumption needs while maintaining a decent quality of life. Because we cannot here address all aspects and sectors of potential activities related to using ICT (e.g., housing, mobility, leisure activities, food), we focus on the general consumption of goods and services.

#### Challenges of unsustainable ICT consumption patterns

The energy and resource demand of ICT significantly depends on user decisions and behavior. Many digital devices have unsustainably short lifetimes, due not only to hard- and software dysfunctionalities but also to psychological or fashion-related obsolescence [126, 127]. Consumers who react to advertisement and business models based on fast product cycles additionally enforce this dynamic [128, 129]. At the same time, repair and maintenance practices remain a niche sector [130]. Even if the most energy efficient devices (efficiency strategy) are bought, data-intensive online activities such as video streaming greatly increase energy use [100, 125]. Users are often unaware of their online activities’ environmental impact because the virtualization of products and services makes energy and material use less visible and creates psychological distance [131]. Online platforms (e.g., social media, streaming services) often rely on business models that generate profit through maximizing user attention and data traffic; hence, frequently using such platforms and services often boosts data. Strategies to increase efficiency can help flatten the curve of rising electricity demand from end users. For instance, moderate scenarios predict steady or even decreasing ICT-related electricity demand due to the rising efficiencies of end-user devices [e.g., 132]. However, for several applications, rising demand for cloud-based apps and services is increasingly shifting end-user-borne electricity demand from the end users’ energy bills to those of providers [133]. Scenarios predict rising absolute energy demand for data centers and telecommunication networks due to cloud services [e.g., 134].

Energy and resource demands of sectors such as mobility or housing or the general consumption of goods and services are increasingly influenced by ICT applications and ICT use patterns [135], and the ubiquitous availability of goods and services in online shops may reinforce high demand levels [136, 137]. When surfing the web, users are exposed to personalized advertisement, which may increase sales more effectively than traditional advertisement [138, 139]. Consumption may also increase due to induction effects if the availability of new appliances stimulates consumption [4]: Røpke et al. [140] early on found that adoption of new ICT or smart household devices increases household electricity use and the resource demand of the devices [see also 141]. Practices within the household are transformed due to a higher level of electrification, and with it the perceived “normal” or “needed” electricity use rises. Consequently, a number of ICT appliances increase consumption, not least due to commercial interests.

These effects, and because the driving force behind most of these challenges is to increase consumption, mean that strategies for efficiency and consistency will probably not suffice to significant reduce the energy and resource demand related to consumption. On the contrary, making end-use applications and devices more efficient and lowering consumption barriers through efficient online shopping, which can save time and money and raise comfort, may even increase demand levels [142, 143]. Hence, promoting user sufficiency also serves as a counterforce to tackle consumption-related rebound and induction effects.

#### Elements of user sufficiency

In light of the challenges described, user sufficiency should be established both for the direct use of ICT devices and for when ICT applications support (or undermine) sufficiency-orientated behavior in general. For the direct use of ICT, a prime sufficiency-oriented behavior is, first of all, questioning whether a digital device is necessary at all—and if yes, purchasing fewer end-user devices and prolonging their service life. Prolonging service life includes users engaging in practices of care, maintenance, and repair. Similarly, practices of sharing, letting, and second-hand acquisition can reduce product purchase. When devices are acquired, their capacity and size should not exceed those capacities actually needed to provide the desired functions. In addition, users may actively decide to refrain from using ICT in certain cases, e.g., by pursuing low-tech practices that hardly consume energy or resources.

To support the interplay between ICT applications and sufficiency-orientated behavior in general, users can apply ICT with the intention of living a less resource-intensive lifestyle [144, 145] or of engaging in citizen behavior for a more sufficiency-oriented society (e.g., [146]). Users can use apps that provide knowledge about sufficiency options [11], such as online tutorials, footprint calculators [147], barcode scanning, or multimodal mobility apps [148]. Some innovations replace energy-intense practices by sufficiency-oriented ones: connectivity, computer-centered work, and video conferencing enable remote work and, thus, can help reduce energy-intense travel, which could be advance by legislation on a “right to home office” [112]. This contribution to reducing energy use applies to hardware but also to numerous other (non-IT) products and services—from sharing rides to sharing software and knowledge. In particular, peer-to-peer sharing platforms can facilitate collaborative consumption [150]. Other appliances facilitate political participation, networking, and organizational tasks for environmental activism (e.g., mailing lists, online petitions, crowdfunding).

#### Policies that promote user sufficiency

Policies targeting user sufficiency should address users directly, as private users, civic actors, and employees at the workplace. But they should also address institutions that influence user behavior, such as e-commerce and social media providers, software developers, and public or civic institutions.

To address users directly about the use of ICT devices, informational and educational campaigns can increase knowledge of and awareness for the environmental impacts of hardware and software. For example, ingredient lists for hardware products can inform consumers about the amount and origin of used resources and enable them to consider corresponding purchase criteria; another example would be a notification on energy use implemented on streaming platforms. Sustainability communication can foster social norms and a consumption culture that make certain practices more attractive, such as buying long-lasting devices, maintaining, caring, and repairing existing hardware. For instance, policymakers as well as employers can promote the idea of “one person—one device”: Since digital devices are multifunctional, users could be encouraged to use one of them as their all-purpose device.

In the realm of ICT for the general consumption of goods and services, sustainability communication can enhance “digital literacy” on issues such as data protection and tracking by operating systems and apps, online shops, and platforms. Likewise, digital literacy regarding sustainable online environments can be fostered to reduce psychological distance to the negative consequences of consumption. Moreover, information on specific apps that help monitor individual footprints (e.g., carbon calculator), test and recommend sustainable products (e.g., barcode scanning apps), or help to improve user skills (repairing, etc.) can be gathered and included in school curricula and consumer guides. In general, sustainability communication can promote the choice of data-secure apps or apps that foster sufficiency-oriented lifestyles. Promotion of relevant gamification apps, social media groups, or online pledging campaigns may further motivate sufficiency-oriented behavior [151, 152].

Communication policies should be accompanied by binding regulations, even if currently, for the direct use of ICT, limited options appear possible and politically acceptable. For instance, personal carbon trading may be an option to reduce the overall footprint of lifestyles, including the ICT footprint, in absolute terms [153] while higher electricity or carbon taxes could provide general incentives to use ICT frugally. Furthermore, in this realm, strict data protection can help reduce data traffic and prevent consumption-stimulating cues. For instance, privacy settings, tracking, and other forms of data collection can be regulated to allow an opt-in instead of an opt-out strategy and to only collect the personal data needed for a certain service. These regulations would also help reduce data traffic.

Further policies relate to online advertising, automated recommendations systems, and incentivizing measures. For online advertisements, software developers of web browsers could be required to set mechanisms to block advertisement by default (again, allowing consumers to opt-in if desired), and platforms and website providers could be forced to allow users who run add blockers. As a more far-reaching measure, a selective advertising ban on certain parts of the Internet (e.g., on search engines, social media platforms) would decommercialize the Internet and, as a co-benefit, also reduce data volume [2, 123]. For automated recommendation systems, providers of such systems, including search engines and advertisers, should be required to publish algorithms and explain inherent preferences comprehensibly so as to improve user understanding of the logic behind (or manipulation of) them. Moreover, regulatory approaches can oblige online shops and market places to provide environmentally relevant product information and to consider sufficiency criteria in their algorithms, search results, and recommendations [44]. For incentivizing measures, platforms can be actively supported to include sufficiency-promoting tools, for instance, by criteria being developed for a sufficiency label. Tools found to be effective can then be integrated into guidelines for mainstream retailers, such as default and filter functions for sufficiency-oriented services in recommendation agents.

### Economic sufficiency

Increasing economic *efficiency* would aim at—in the sustainability context of this article—either achieving a given economic output with the lowest possible energy and resource input or maximizing economic output with limited resource and energy input. For instance, Industrial Internet of Things platforms and smart factories can contribute to reducing the relative resource and energy input of industrial production per unit of output [154–156]. Strategies aiming at economic consistency want to establish a Circular Economy, which—besides sourcing recycled or renewable resources (see hardware consistency)—requires overarching measures that align production cycles with natural cycles, including nature-based solutions [26, 157, 158].

In contrast, *economic sufficiency* aims at digitalization supporting a transition to an economy characterized not by economic growth as the primary goal but by production and consumption sufficient to serve existing societal and individual needs. To this end, it empowers sufficiency-oriented business models that focus on nurturing public and common goods rather than those striving for market share and capital accumulation.

#### Challenges of ICT fostering a growth-oriented economy

ICT application is deeply entwined with recent developments of the global economy, including pressures on energy consumption, resource extraction, and greenhouse gas emissions. ICT foster economic growth through three mechanisms. First, ICT are used to rationalize employment and thereby increase labor productivity [159, 160]. As empirics show, improved labor productivity arising from digital technologies is turned into an expansion of production [85, 161]. Second, ICT may facilitate improvements in energy and resource efficiencies [3, 63]. In the first instance, this facilitation is beneficial for the environment as it decreases environmental intensities. At the same time, energy efficiency improvements lead to economy-wide rebound effects, implicating economic growth and, at least, a partial offsetting of any potential energy savings [54, 162]. While empirical investigations are hard to come by, the vast increases in ICT energy efficiency and their immense growth over the last years suggest high rebound effects [9, 12, 15]. Third, ICT enable product and service innovations, including numerous new ICT devices. These innovations generate new markets and new consumption potential [20]. Empirical evidence suggests that digitalization has so far been accompanied by stronger economic growth [87, 163, 164] and increasing electricity consumption [165–167], while findings for digitalization’s impacts on CO2 emissions are mixed [86, 166, 168–170].

The described interrelationship between ICT, economic growth, and environmental impacts depend on historical and country-specific circumstances. Most importantly, they depend on which actors develop and disseminate the ICT and under which economic circumstances this development takes place [2]. Today, most ICT hardware and most apps, platforms, and webpages with large amounts (billions) of users are designed by large global shareholder companies, including, and most notably, the so-called “GAFAM” group (Google/Alphabet, Apple, Facebook, Amazon, Microsoft) as well as Tencent, Alibaba, and others in the Chinese market. Moreover, many digital innovations are financed by high levels of venture capital. Accordingly, many new digital services are primarily tailored to deliver high return on investments and high dividends.

In addition, large IT companies such as Alphabet or Facebook make major revenues from commercials [171]. Addictive designs are one strategy to continuously increase these revenues [172]. Amazon’s business model entails high sales figures and growing digital services such as cloud computing [173]. And hardware suppliers, such as Apple, Huawei, Samsung, Sony, and others, focus on speedy product cycles to increase sales [174]. Hence overall, large parts of the development of new ICT devices and services are tailored towards growth-oriented business models—rather than towards sustainable production and consumption patterns.

#### Elements of economic sufficiency

To achieve economic sufficiency, ICT-borne improvements in labor productivity should be used not to foster economic growth but to reduce average working hours, leaving more time for care work, repairing, or (urban) subsistence [175]. ICT technological possibilities must be primarily used to increase resource and emission efficiencies and foster circular economies rather than focusing on increasing labor productivities. By preventing rebound effects through appropriate policies (see below), a strategy towards economic sufficiency will ensure that the economic saving potentials from efficiency improvements is not turned into economic growth [176].

Different types of economic organization are required for an alternative technological development towards resources rather than labor productivity and towards sufficient production rather than continuous economic growth [177, 178]. Resultingly, crucial pillars of economic sufficiency are various types of non-profit-oriented, peer-to-peer-organized, stakeholder-driven, and/or collectively owned firms and organizations [179]. Numerous business cases exist, but many of them are smaller and less influential than the large-scale shareholder-driven platforms and businesses. Examples of digital non-profit organizations are Wikipedia or, as a purpose-driven business model, the search engine Ecosia. Getaround or SnappCar are examples of peer-to-peer-organized platforms, e.g., for mobility, that contrast to growth-oriented platforms such as Uber. Examples of platforms cooperatives [180] are the Trans Union Car Service or Union Taxi. Comprehensive policies and measures are needed that help those actors leave the niche and scale up to become dominant platforms in the Internet.

#### Policies that promote economic sufficiency

Policies targeting economic sufficiency mainly address policymakers at the federal or communal level in the fields of economic and labor policy, environmental policy, and firm regulation.

To support economic sufficiency, governments need not only to change regulations incrementally but, much more fundamentally, to shape the digital economy to make it sustainable [181, 182]. In particular, prices, incentives, infrastructures, and public funding need to allow and enable all economic actors to act sufficiently.

Improved labor productivity due to ICT application can be met with reduced average working hours [183, 184]. These can be initiated by incentives and regulations. The most well-known approach is limiting the maximum number of working hours in a country or in a sector. Increasing importance can also be gained through maternity and parental leave. “Time rights” would give individuals the right to reduce working time—be it per day, week, month, or year [185]. Central actors are not only governmental institutions but also trade unions and employers’ associations [186]. Moreover, wage policies (e.g., raising of minimum wages) could ensure that productivity improvements are turned into higher wages and, hence, support steady wages even when working hours are reduced. Additional labor policies can improve reorganizing work to flexibly tailor it to employees’ needs [81].

Rebound effects can be counteracted by increasing the costs of energy consumption. Policies include either taxes, on CO2 emissions, electricity, and other energy carriers, or emission trading systems [54, 187]. As suggested by Weizsäcker et al. [188], taxes can increase the cost of energy *in parallel* to increasing energy efficiencies so that energy service costs stay constant. As a result, incentives to continuously increase efficiency are maintained while an efficiency-induced expansion of production and consumption is prevented. At the same time, taxes on labor can be reduced so that employment is supported and labor-intensive technology becomes more competitive vis-à-vis automation/robotization. Taxes on labor would also incentivize sufficiency practices and business models, e.g., repairing and handcraft. Alternatively, new levies, such as a “robot tax,” have been discussed [189].

Regarding the firm level, first of all monopoly and competition law must be adapted to adequately address power asymmetries in digital markets [2]. Moreover, the playing field between smaller companies and large shareholder companies can be levelled by making common interfaces obligatory for social media and other platforms, as was instigated for email providers years ago. To reduce data transmission and collection with a view both to reducing environmental footprints and conforming with privacy regulation implies a deep restructuring of business cases that base their profits on selling data and data-based advertisement. With a view to social media providers, strict data protection would help to divert interest from “economy of attention”-business models that rest on generating and commercializing user data. To reduce attention-grabbing practices, push messages and notifications should only be allowed by opt-in.

Internet platforms that function as “quasi-natural” monopolies due to large-scale network effects could either be communalized [18] or legally bound to cooperative business models. Such platform cooperatives, in turn, can be supported by developing appropriate software and building an ecosystem for platform cooperatives [180]. For instance, communal governments could foster development of cooperative mobility as a service-platforms (MaaS) or e-commerce platforms for local businesses. National governments could provide further public funds specifically addressing the founding of platform cooperatives. Moreover, procurement regulations can be adapted so that local authorities give preferential treatment to goods and services offered by platform cooperatives.

## Discussion

Our analysis indicates the importance of not solely focusing on strategies that improve efficiency and consistency but of shifting the focus towards total environmental impacts by means of digital sufficiency. While efficiency often relies on technological progress and consistency relies on reorganizing the production process, sufficiency is more connected to human and societal behavior and to the consumption side. Only by focusing on total environmental impacts can measures for environmentally sustainable ICT use be strongly connected to boundaries, such as planetary boundaries, which must be set by, or at least reflected in, political interventions.

### Interrelations of the four dimensions

**Fig. 1:**
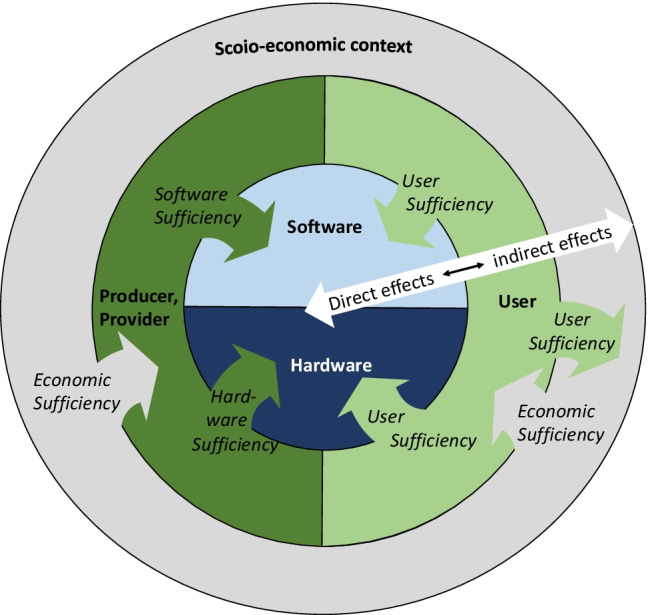
Interplay of the four dimensions of digital sufficiency

As Fig. 1 shows, the dimensions of digital sufficiency align in concentric circles from direct effects over indirect effects to general economic framework conditions. The first two dimensions of the digital sufficiency that we define—hardware and software sufficiency—are related to the first-order effects (“direct effects”) of digitalization. Hardware and software sufficiency measures aim at reducing the total environmental impact throughout the life cycle of ICT hardware and at developing software that is not driving increased data traffic and electricity use of networks. Some elements of user sufficiency are directly related to Hardware and software sufficiency, e.g., the data traffic that users generate by their activities. Reduced traffic by, e.g., not streaming unnecessary amounts of HD videos would reduce the demand for hardware while also reducing operational energy. Those three elements—hardware sufficiency, software sufficiency, and user-generated traffic—have in common the relation to the first-order/direct effects of ICT.

Other elements of user sufficiency, i.e., the use of ICT for sustainable lifestyles, as well as the dimension of economic sufficiency relate to indirect effects. They address creating opportunities for users and producers to limit society’s total resource use as a whole, i.e., in sectors beyond ICT (see Fig. 1).

Altogether, the direct and the indirect implications of digitalization can develop either towards increasing or towards decreasing environmental burdens. As we have discussed in Section 2.2., focusing on most efficient ICT, on making hardware production more circular, and on running ICT on renewable energies may not ensure that ICT have lower net energy and resource use. The implications of digitalization on wider society are likely to be more important than the effects from the direct use of the technology [68]. The four dimensions of the concept of digital sufficiency provide a comprehensive perspective that considers digitalization’s implications on total resource and energy use in society and how it can be used to reduce these in absolute amounts.

### Strengths and limitations of policies for digital sufficiency

To implement the policies suggested with regard to the concept of digital sufficiency, concerted actions from multiple actors are crucial. Software developers and companies can create ICT products and business models that not only enable hardware sufficiency and software sufficiency but also support user sufficiency and economic sufficiency. Civil society actors can demand guidelines and activate citizens. Of utmost importance for digital sufficiency, however, are effective actions by policymakers at various levels. Table 1 summarizes key policy measures suggested for each subdimension, grouped according to respective societal actors.

**Table 1:** Measures to strengthen digital sufficiency

	Dimension: societal actor:	Hardware sufficiency	Software sufficiency	User sufficiency	Economic sufficiency
	Definition	Producing & designing hardware for longevity, repairability, and with the least possible resource and energy demand	Software development and implementation that ensures long-term functionality and the lowest possible data traffic and hardware utilization for task performance	Users apply digital devices frugally and make use of ICT in a way that fosters sufficiency-oriented lifestyles	ICT-borne improvements are used to nurture public and common good instead of economic growth
A c t o r s	Producer/developer (from mining to fabrication)	• Production of long-lasting and repairable devices • Share blueprints of devices • Increased share of recycled materials and reused parts • Mandatory reporting on production conditions • Reporting on materials used for components	• Provide software upgrades to ensure long use of hardware • Design appliances with minimal data collection and computation; ensure long-term compatibility with hardware, e.g., by not requiring more storage space • Provide open source software, or at least open standards, and ensure backward compatibility • Provide default settings favoring minimal energy demand, limiting the extent of forced connectivity		
	Seller, provider (incl. Data Center Provider)	• Promotion of sufficient use: avoiding contracts including phones to prolong use phase, offer repair and maintenance services • Letting instead of selling devices (device-as-a-service)	• Provide open source software • Avoid business models based on commercial data use • Make use of Demand-Side-Management measures for grid-balancing purposes • Use IT management software in data centers • Default settings for minimal data use, e.g., strict privacy settings, minimal resolution for online video, erasing unnecessary data, disabling autoplay	• Only collect personal data essential for each specific service • Block advertisement by default	
	Private user	• Buy long-lasting, smaller and fewer devices; maintaining, caring and repairing to prolong lifetime • Return devices to formal collection points	• Choose providers that perform sustainably (e.g., Nextcloud)	• Choose data-secure apps, use apps that foster sufficiency-oriented lifestyles	
	Organizational user	• Buy long-lasting, smaller and fewer devices • Establish maintenance services for caring & repairing to prolong lifetime	• Apply criteria of energy efficiency, sufficiency and data-security when choosing appliances		
	Policy regulators	• Set minimum social and ecological standards for resource extraction and production (Green IT) • Set standards for reparability, upgradability, and compatibility • Introduce mandatory recycling quotas • Set standards for absolute energy consumption of hardware • Develop pricing policy for data centers that incentivize low energy demand	• Extend the European Ecodesign Directive to software products • Regulation for maximum energy use per transaction	• Regulation for data collection, security, tracking, privacy; open data policies • Mandatory publishing of preferences for automated decision making (ADM) systems • Introduce an opt-in instead of an opt-out strategy (privacy settings, tracking and other forms of data collection) • Higher electricity or carbon taxes	• Adapt monopoly and competition law to adequately address power asymmetries in digital markets • Introduce taxes, subsidies or incentives, infrastructures and public funding
	Civil society	Activism and political participation that demand sufficiency-oriented production, consumption, data handling, and legislation; grassroot movements, associations, voluntary work (e.g., repair café)			

For the four dimensions of digital sufficiency, both the challenges for comprehensive political implementation and the effectiveness of the measures appear to increase with each dimension. For the first dimension, hardware sufficiency, it seems comparatively feasible to implement the suggested policies. These policies also have many similarities with existing policies from other domains, for example, design directives for other electronic devices, recycling quotas for bottles, or energy and off-heat use-standards for buildings. Still, some hardware sufficiency policies would require more juridical investigations and would probably face more resistance from producers, e.g., legislation for a right to repair devices.

In comparison, implementing the suggested policies for software sufficiency appear to require more research and can draw less on policy experiences from other domains. For instance, developing energy standards for software products requires developing new criteria and standards and also new ways of measuring and monitoring. Likewise, extending existing design directives to software products is more complicated than including ICT hardware products into existing product catalogues. Moreover, suggestions such as an advertising ban on selected Internet areas (e.g., on search engines, social media platforms) could face significant political opposition from IT companies or from the entire marketing industry. However, given that the share of energy use from applying ICT throughout economy and society is higher than the energy consumed in producing hardware [76, 103], comprehensive measures for software sufficiency would also contribute more to reducing the overall burden from the sector.

Challenges—but also effectiveness—increase further when looking at user sufficiency. Endeavors to suggest price incentives that are truly “felt” by consumers, such as high electricity prices or binding legislation on personal carbon trading, are notoriously difficult to implement. Therefore, most policies suggested for user sufficiency in this paper comprise (a) user-targeted communication activities that aim to enhance digital literacy, repair skills, knowledge about environmental impacts, etc., and (b) legislation for producers and platforms, including, for instance, strict privacy regulations that foil personalized advertisement or legislation requiring product sustainability information. However, while strict standards for producers or platforms can be effective, they are not suited to addressing potential rebound effects on the user side. At the same time, communication and education activities are comparatively weak instruments and may not fully prevent unsustainable use patterns. However, some measures suggested for hardware and software sufficiency, such as increasing the durability of devices or offering services requiring low data processing and transmission, can effectively reduce the size of rebound effects caused by users.

Finally, some of the measures suggested for economic sufficiency may be even more difficult to put into practice. Changing prices, labor market policies, infrastructures, and public funding to reduce necessity for, and reliance on, economic growth stretches far beyond the question of ICT governance. For instance, while an Ecological Tax Reform with continuously increasing tax rates on energy or emissions may be well suited to addressing economy-wide rebound effects, recurring discussion on only minor increases in such tax rates show how difficult its implementation is in “realpolitik.” Incentive measures to support platform cooperatives or projects that pursue public platforms may be more easily implemented. Still, the overall aim of the subdimension of economic sufficiency to support a transition to an economy characterized not by economic growth as the primary goal but by sufficient production and consumption [191] not only requires much more vigorous research but remains uncertain with a view to its political implementation. If, however, such a shift could be realized, its effectiveness in reducing energy and resource demand, as well as related emissions, would probably be even higher than that of measures associated with the other three dimensions of digital sufficiency—as the coupling of reduced economic output and demand shows in times of breakdowns, such as during the Corona pandemic.

## Conclusion

While research on the environmental implications of ICT becomes increasingly complex and interdisciplinary, large research gaps remain. For instance, the current state of research is still far from being able to draw a line and postulate whether the aggregated net effect of introducing digital technologies and services into society reduces existing environmental burdens or whether it actually aggravates them (see Section 2). Far more systematic knowledge is needed to answer this question.

This conceptual article followed a “precautionary approach” and developed interdisciplinary perspectives and strategies that address some of the driving forces countervailing the savings potential of ICT. Particularly, the article has questioned whether efficiency and consistency strategies suffice to realize a net beneficial contribution of digitalization and suggests that comprehensive policies for digital sufficiency are indispensable if ICT should play an unequivocally beneficial role in the overall environmental transformation.

The concept digital sufficiency constitutes a basis to understanding how ICT can become part of the essential environmental transformation. The four dimensions of digital sufficiency—hardware, software, user, and economic sufficiency—allow for a nuanced and comprehensive view. Based on this view, we have developed a large number of heterogeneous policy proposals and have come to the conclusion that, while policies for hardware and software sufficiency are more easily conceivable and politically realistic, policies for user and, even more so, for economic sufficiency are relatively ambitious. However, the policies for user and economic sufficiency are also similar to the policies for the environmental transformation in general. This similarity shows that digital sufficiency would be part of a greater transition that—albeit difficult to implement—would significantly reduce energy and resource demand and emissions, reductions that are indispensable to remaining within planetary boundaries.

## Footnotes

1 Note that information about software *consistency* (in the sustainability context of consistency as closing material cycles) is not yet available or included here. It may be developed in another context.
